# A Network Analysis of the Relationships Between Behavioral Inhibition/Activation Systems and Problematic Mobile Phone Use

**DOI:** 10.3389/fpsyt.2022.832933

**Published:** 2022-04-01

**Authors:** Lingfeng Gao, Wan Zhao, Xiaowei Chu, Haide Chen, Weijian Li

**Affiliations:** ^1^Institute of Psychological and Brain Sciences, Zhejiang Normal University, Jinhua, China; ^2^School of Psychology, Central China Normal University, Wuhan, China

**Keywords:** behavioral inhibition systems, behavioral activation systems, problematic mobile phone use, network analysis, components model of addiction

## Abstract

**Background:**

It is of great concern to society that individuals can be vulnerable to problematic mobile phone use (PMPU). However, there are a few studies in the field evaluating associations between behavioral inhibition/activation systems (BIS/BAS) and PMPU, and the results have been inconsistent. This study aimed to explore the relationships between BIS/BAS and PMPU by network analysis.

**Methods:**

A total of 891 young adults participated in the study. BIS/BAS and PMPU were assessed by using the behavioral inhibition and activation systems scale and smartphone application-based addiction scale, respectively. The structure of the BIS/BAS-PMPU network was characterized using “strength,” “closeness” and “betweenness” as centrality indices. Edge-weight accuracy and centrality stability were tested using a bootstrap procedure.

**Results:**

The network analysis showed that “mood modification,” “tolerance” and “withdrawal symptoms” had high centrality. In addition, the positive connection between BIS and “mood modification” or “tolerance” and between BAS-fun seeking and “mood modification” or “conflict” were also shown in the BIS/BAS-PMPU network.

**Conclusion:**

These findings shed light on the central and bridge components between the BIS/BAS and PMPU communities, providing new evidence relevant to potential mechanisms that account for how high-BIS or high-BAS individuals develop PMPU, and inspiring component-based PMPU prevention or interventions.

## Introduction

Broadly, problematic mobile phone use (PMPU) refers to an inability to regulate individual proper use of the mobile phone, which eventually involves negative consequences in daily life (1). It shares some key features of addictive behaviors (2), and has also been called “smartphone addiction” (3). In recent decades, PMPU has become a common problem among adolescents and young adults, and there is a burgeoning field of investigation owing to the significance of its consequences. A recent systematic review concluded that there are five categories of risky consequences of PMPU: cognitive control, emotional health problems, physical health problems, professional performance, and social performance (4). What is even more surprising is that individuals with PMPU showed lower brain volume and weaker intrinsic neural activity relevant for craving and inhibitory control (5). In addition, the prevalence of PMPU seems to have increased, especially during or after the COVID-19 pandemic [e.g., (6–9)]. In light of its consequences and prevalence, it is necessary to prevent individuals, especially susceptible people, from being addicted to mobile phones.

Previous studies have indicated that personality traits are associated with PMPU (10–13). The Big Five personality traits are of particular interest, and some of them are related to PMPU [see (14)]. From the perspective of physiological models of personality, some researchers have explored the relationship between behavioral systems and addiction (15–18). However, relatively few studies have focused on such a relationship in the field of PMPU, and the results have been inconsistent. More importantly, the relationships between behavioral systems and PMPU has traditionally been studied utilizing latent class analysis. A few studies, based on a systematic network perspective, have been conducted to understand these relationships, which could better explain the nature of the associations between behavioral systems and PMPU. Specifically, a systematic network perspective emphasizes the relationships between variables in the network system, which is more suitable for real situations (19). In addition, the visualization and dynamic study of the network enables us to more intuitively see the structure and dynamic evolution of the relationships between the behavioral systems and PMPU (19). Furthermore, network analysis can reveal the links between components of behavioral systems and PMPU by statistically punishing false correlations, which may solve the traditional issue of overrating the link strength of behavioral systems and PMPU (20). Finally, network analysis can provide multiple indices of nodes and edges to describe the relationships between behavioral systems and PMPU (19, 21).

### Theoretical Background

#### Components Model of Addiction

Drug and behavioral addiction seem to have many commonalities (22, 23). Based on an eclectic approach to the study of addictive behaviors and the aim of increasing the general public's perception of addictive behaviors, the components model of addiction proposed that addictions need to present six distinct symptoms of behavior or psychological feelings (24). These symptoms of addiction are salience, mood modification, tolerance, withdrawal, conflict, and relapse. Specifically, salience refers to the fact that individual thinking, emotions, and behavior are almost dominated by a particular activity that becomes the most important activity in daily life. Mood modification refers to individual subjective experiences, such as provocation, excitement, or tension, followed by engaging in a particular activity. Tolerance refers to individual gradually increasing their time and investment in a particular activity to get the experience that the individual had before. Withdrawal symptoms refer to individuals tending to have unpleasant feeling states (e.g., extreme moodiness and irritability) and/or physical effects (e.g., nausea, sweats, headaches, insomnia and other stress-related reactions), when particular activities are interrupted. Conflict refers to particular activities that elicit conflicts between the individual and those around them (interpersonal conflict) or within the individual themselves (intrapsychic conflict). Relapse means that although there may be a period of control or abstinence, the particular activity can easily recur, and will show stronger tendencies when it occurs again. PMPU can be viewed from the perspective of this model and might also present these six symptoms.

#### Reinforcement Sensitivity Theory

Reinforcement sensitivity theory [RST (25)] attempts to explain individual differences from the perspective of human neurophysiological mechanisms. The theory suggests that there are some subsystems in the central nervous system that are sensitive to rewards and punishments, and regulate individual emotions and behaviors through reinforcement effects. The behavioral inhibition system (BIS) and behavioral activation/approach system (BAS) are two basic subsystems (26, 27). BIS is sensitive to conditional aversive stimuli. When a signal of punishment or termination of rewards is presented, the system is activated, accompanied by a negative emotional experience and withdrawal/avoidance behavior. BAS is sensitive to conditional appetitive stimuli. When a signal of reward or termination of punishment is presented, this system is activated to generate a positive emotional feeling and approach behavior. BAS consists of drive (BAS-D), reward responsiveness (BAS-R) and fun seeking [BAS-F (28)]. BAS-D refers to engaging strong and quick goal pursuit. BAS-R refers to receptivity to reward. BAS-F refers to the desire for new and potentially rewarding experiences. Because BAS is not a unified construct in relation to psychopathology, it is important to focus on each component of BAS carefully (29, 30).

The relationships between BIS/BAS and addiction have been supported by some evidence in the field of drug addiction. It was found that high reward (i.e., BAS) and low punishment sensitivity (i.e., BIS) were significantly related to drinking habits (31) Kambouropoulos and Staiger (32) also found that a higher BIS was associated with alcohol abuse. In addition, studies have shown that individuals with drug addictions had higher BIS and BAS-F scores than controls (15, 33).

### Associations Between BIS/BAS and PMPU

With the rapid growth of network technology, problematic internet use behavior is becoming prominent (34–36). Researchers have started to pay attention to the causes of problematic internet use behavior. The relationships between BIS/BAS and problematic internet use behavior have captivated researchers' attention in recent decades. A recent study found that the BIS and BAS scores of people with internet gaming disorder (IGD) or internet addiction (IA) were higher than those of non-addicted people (37). Regarding specific dimensions of the BAS, BAS-F seems to have a stronger relationship with IA. Hierarchical regressions showed that the BIS emerged as a positive predictor of IA and that only BAS-F among the BAS components positively predicted IA (38). Individuals with IGD have higher BIS and BAS-F scores than non-IGD individuals (39). High BAS-F and high BIS scores have positively correlate with the severity of IA (18). A 1-year follow-up study has provided reliable evidence and revealed that individuals with higher BAS-F scores were more likely to develop IA (40). Similar results were also observed among adolescents with attention-deficit/hyperactivity disorder (41). Summarizing the above, it seems that an individual with high BAS-F or BIS scores was prone to IA. However, there have been inconsistent results. For example, some studies failed to observe that the BIS or BAS was related to IA (39, 42, 43). Regarding the specific dimensions of the BAS, low BAS-D scores were associated with severe IA (41). Another study showed that only BAS-D was a risk factor associated with IGD (44). Furthermore, there was a significant negative correlation between IA and BAS-R among high school students (39).

Recently, emerging studies have started to explore the relationships between BIS/BAS and PMPU. However, the number of relevant studies remains insufficient and the results have been mixed. To our knowledge, there are only four studies evaluating the relationship between BIS/BAS and PMPU. Kim et al. (45) found that BAS-D and BAS-R, not BAS-F and BIS, were predictors of PMPU. Jiang and Zhao (46) found that BIS was negatively correlated with PMPU. However, BAS was positively correlated with PMPU. Lee et al. (47) only reported BIS results and found a positive association between BIS and PMPU. Jeong et al. (48) found that the PMPU group had higher BIS and BAS scores than the normal group.

At present, these results are relatively confusing, which may be due to studies used different PMPU scales, and these different scales incorporated different symptoms of addiction. Thus, the BIS/BAS may be linked not only to PMPU, but also to the different symptoms of PMPU. In addition, many studies have not further analyzed the relationship between the dimensions of the BAS and PMPU, and therefore, the relationship between the BAS and PMPU have not been clarified.

### Network Analysis

The method of network analysis involves presenting the characteristics and information of a system in interconnected network form. The system is composed of “nodes” and “edges” (49, 50). Nodes represent psychological variables such as mood states, symptoms, or attitudes, while edges (i.e., the links connecting two nodes) represent unknown statistical relationships that need to be estimated (20). Network analysis focuses on the relationship between variables and highlights their important aspects through statistical modeling. Therefore, network analysis can reveal the data patterns that are difficult to see in the latent variable model.

Some scholars have carried out research on PMPU by utilizing network analysis, with a focus on three aspects: the symptoms, associated comorbidities, and influencing factors of PMPU. First, regarding the symptoms of PMPU, Huang et al. (51) explored the PMPU network and found that loss of control was the key symptom. Another study found that compulsive use had higher centrality levels in the PMPU network (52). Second, regarding the comorbidities associated with PMPU, a recent study conducted an exploratory graph analysis and showed that PMPU and problematic WhatsApp use were heavily intertwined (53). Last, regarding the influencing factors of PMPU, Wei et al. (54, 55) explored network pathways and further discovered the “central” components and the “bridge” components between neuroticism and PMPU communities. Huang et al. (56) employed a network analysis approach to understand the interaction between PMPU and related influencing factors and found that there were several central influencing factors (such as self-control ability, loss of control, parent-child relationship, and peer attitudes toward smartphone use) and bridge factors (such as peer attitudes toward smartphone use, peer pressure for smartphone use, and fear of missing out).

Psychopathological network theory portrays mental disorders as causal systems of interacting symptoms (57). Symptoms cease to be passive indicators of the underlying common cause of illness and become dynamic components of the system (21). To our knowledge, there has been a lack of research on the symptoms of PMPU based on the addiction components model and on the relationship between BIS/BAS and PMPU by utilizing network analyses. This lack of research might obscure meaningful associations between BIS/BAS and addictive symptoms of PMPU and make it difficult to identify individual symptoms that may be more critical to the onset or maintenance of PMPU.

### The Present Study

Through the above literature review, we identified the following limitations in the current research on the relationships between BIS/BAS and PMPU: First, the results describing the relationship between BIS/BAS and PMPU have been mixed and further clarification is needed. Second, the relationship between BIS/BAS and specific addictive symptoms of PMPU have not been explored. Finally, many studies have used latent variables for these analyses, which ignore the network structure of the relationship between variables. To bridge these gaps, this study attempted to explore the relationship between BIS/BAS and PMPU by using network analysis. We posed the following research questions:

R1: Which edges play the role of bridging the BIS/BAS community and PMPU community?

R2: Which symptoms are central in the BIS/BAS-PMPU network?

## Methods

### Participants and Procedure

The ethics of all procedures in the research were approved by the ethics committee of the authors' research institution. A total of 914 university students from two provinces in China participated in the study by utilizing convenience sampling from December 2019 to January 2020 (before the COVID-19 epidemic). The survey was conducted by using the WeChat-based Wenjuanxing program. All participants received instructions and were told that their participation was voluntary and they could withdraw from the study at any time for any reason. To encourage honest responses, the anonymity of the survey was emphasized. Participants who met the following criteria were included: (1) ability to understand Chinese; (2) consent to participate in the study; and (3) owned a mobile phone. We eliminated twenty-three participants who (1) completed the surveys, but indicated they did not respond at the appropriate times or (2) responded improperly or randomly on some of the essential study variables. The final sample was 891 students. Across the entire sample, 218 of the participants were men (24.47%), and 673 were women (75.53%); 485 were from rural areas (54.43%), and 406 were from urban areas (45.57%). The mean age of the participants was 19.09 years (*SD* = 1.27; range: 16–24 years).

### Measurements

#### Smartphone Application-Based Addiction Scale

The smartphone application-based addiction scale (SABAS) is the most recently developed scale for assessing PMPU (2). The Chinese version of the scale, revised by Leung et al. (58), was used in this study. The scale was developed based on the addiction components model (24) and contained six items, such as “Over time, I fiddle around more and more with my smartphone,” that corresponded to addiction components (i.e., salience, conflict, mood modification, tolerance, withdrawal symptoms, and relapse). A 6-point Likert-type scale from “1 = Strongly disagree” to “6 = Strongly agree” was used. Higher scores on the SABAS related to greater risk of PMPU. Cronbach's α coefficient was 0.76 in our sample.

#### Behavioral Inhibition and Activation Systems Scale

The BIS/BAS scale was utilized to assess sensitivity to punishment and rewards (28). The Chinese version of the BIS/BAS scale was revised by Li et al. (59), which deleted the items “Even if something bad is about to happen to me, I rarely experience fear or nervousness” and “I have very few fears compared to my friends” considering the low item discrimination of the two in Chinese sample. The Chinese version of the scale has 18 items in total. The BIS scale contained 5 items (e.g., “If I think something unpleasant is going to happen. I usually get pretty ‘worked up”'). The BAS scale consisted of 13 items, “If I think something unpleasant is going to happen, I usually get pretty ‘worked up”' (BAS-R; e.g., “When I get something I want, I feel excited and energized”), 4 items assessing drive for goals (BAS-D; e.g., “I go out of my way to get things I want”), and 4 item assessing fun-seeking (BAS-F; e.g., “I will often do things for no other reason than that they might be fun.”). The Cronbach's α coefficients of the BIS, BAS, BAS-R, BAS-D and BAS-F were 0.82, 0.85, 0.85, 0.74, and 0.73, respectively, in our sample.

### Statistical Analysis

Descriptive statistics, Pearson correlations, and Cronbach's α were performed by using SPSS 25. Cohen's *d* and network analysis were conducted by using JASP (Jeffrey's Amazing Statistics Program). Edge-weight accuracy and centrality stability were tested by using R (Version 4.1.2).

A visualized network analysis was performed in the present study. A graphical LASSO [glasso (55)] estimated least absolute shrinkage and selection operator (LASSO) regularization. A regularized Gaussian graphical model [GGM; (60, 61) was estimated by utilizing glasso in combination with the extended Bayesian information criterion [EBIC (62)] model (i.e., EBICglasso model). The EBICglasso model was calculated by using JASP. The tuning parameter was set to 0.5 for a more parsimonious and easily explainable network (i.e., fewer edges, higher specificity and sensitivity). The connection of nodes was described using edges. Edge thickness was used to indicate the strength of the association between nodes. Blue edges indicate positive correlations and red edges indicate negative correlations. The centrality of nodes in the network, which represented study variables, were calculated using measures of betweenness (degree of connectivity), closeness (distance centrality), and strength [degree centrality (63)]. Centrality measures are reported as standardized values (*z-*scores).

Edge-weight accuracy and centrality stability were used by the R-package bootnet (Version 1.4.3). Bootstrapped 95% confidence intervals were utilized for estimating the accuracy of edge-weight (the number of bootstrap samples was 1,000) and fewer overlaps indicated higher stability/accuracy. The stability of node attributes was estimated by using the case-dropping bootstrap procedure. If most samples can be excluded from the dataset without observing significant changes in the centrality index of the node, the network is considered to be stable. Centrality stability is represented graphically and quantified by calculating the correlation stability coefficient (*CS*-coefficient), which was suggested to preferably be above 0.5 and should not be below 0.25 for interpretability and stability (49). The small-worldness index (SWI) was calculated by considering both the average shortest path length and the global aggregation coefficient, SWI = (C/C') / (L/L'), where L and C are the shortest path length and the global aggregation coefficient of the network, and L' and C' are ER random networks [Erdos-Rényi model (64)] with the same number of nodes and connections. If the SWI is >1, it means that the network has small-world characteristics, i.e., high connection strength, short average paths between nodes, and strong overall connections (57).

## Results

### Descriptive Statistics and Correlation Analyses

Sample characteristics and group comparisons for the study variables are shown in Table 1. The PMPU, BIS and BAS-R scores were significantly different by sex. Women all had higher PMPU, BIS and BAS-R scores than men. In addition, the BAS-D and BAS-F scores were significantly different by residence. The participants from urban areas had higher BAS-D and BAS-F scores than the participants from rural areas.

**Table 1:** Sociodemographic characteristics.

		***n*** **(100%)**	**PMPU**	***t***	***d***	**BIS**	***t***	***d***	**BAS-R**	***t***	***d***	**BAS-D**	***t***	***d***	**BAS-F**	***t***	***d***
			***M*** **±** ***SD***			***M*** **±** ***SD***			***M*** **±** ***SD***			***M*** **±** ***SD***			***M*** **±** ***SD***		
Gender	Male	218 (24.47%)	3.48 ± 0.92			2.86 ± 0.48			3.12 ± 0.49			2.85 ± 0.51			2.79 ± 0.48		
	Female	673 (75.53%)	3.66 ± 0.88			2.97 ± 0.48			3.21 ± 0.46			2.80 ± 0.50			2.79 ± 0.49		
				2.49*	0.20		3.06**	0.23		2.40*	0.19		1.19	0.10		0.12	0.00
Residence	Rural areas	485 (54.43%)	3.58 ± 0.87			2.94 ± 0.47			3.17 ± 0.47			2.77 ± 0.47			2.75 ± 0.49		
	Urban areas	406 (45.57%)	3.65 ± 0.90			2.94 ± 0.49			3.22 ± 0.48			2.87 ± 0.53			2.84 ± 0.48		
				1.18	0.08		0.04	0.00		1.52	0.11		2.80**	0.20		2.77**	0.19
Only child	Yes	365 (40.97%)	3.56 ± 0.85			2.93 ± 0.46			3.17 ± 0.48			2.85 ± 0.51			2.78 ± 0.50		
	No	526 (59.03%)	3.66 ± 0.92			2.95 ± 0.49			3.20 ± 0.46			2.79 ± 0.49			2.80 ± 0.48		
				1.64	0.11		0.44	0.04		0.92	0.06		1.88	0.12		0.45	0.04

* *<0.05*,

** *<0.01. PMPU, Problematic mobile phone use; BIS, Behavioral inhibition systems; BAS-R, Behavioral activation systems-reward responsiveness; BAS-D, Behavioral activation systems-drive for goal; BAS-F, Behavioral activation systems-fun seeking*.

Descriptive statistics and correlation analyses for the study variables are shown in Table 2. PMPU was significantly positively associated with BIS (*r* = 0.22, *p* <0.001) and BAS-F (*r* =0.20, *p* <0.001) scores. Similarly, the six components of PMPU were all significantly positively associated with BIS and BAS-F scores and the correlations among them ranged from small to medium. In addition, BAS-R scores were significantly positively associated with salience (*r* =0.07, *p* =0.048) and mood modification (*r* = 0.12, *p* < 0.001) scores.

**Table 2:** Correlation analysis of the study variables.

	***M*** **±*****SD***	**1**	**2**	**3**	**4**	**5**	**6**	**7**	**8**	**9**	**10**
1 PMPU	3.62 ± 0.89	-									
2 Salience	3.94 ± 1.33	0.63***	-								
3 Conflict	3.37 ± 1.48	0.61***	0.20***	-							
4 Mood modification	4.11 ± 1.26	0.68***	0.37***	0.31***	-						
5 Tolerance	3.84 ± 1.27	0.73***	0.38***	0.26***	0.42***	-					
6 Withdrawal symptoms	3.13 ± 1.29	0.76***	0.34***	0.40***	0.40***	0.45***	-				
7 Relapse	3.30 ± 1.25	0.68***	0.28***	0.24***	0.29***	0.50***	0.51***	-			
8 BIS	2.94 ± 0.48	0.22***	0.10**	0.13***	0.23***	0.20***	0.12***	0.13***	-		
9 BAS-R	3.19 ± 0.47	0.05	0.07*	0.01	0.12***	0.06	−0.04	0.01	0.44***	-	
10 BAS-D	2.81 ± 0.50	−0.02	0.06	−0.02	0.003	−0.06	−0.03	−0.03	0.11**	0.47***	-
11 BAS-F	2.79 ± 0.49	0.20***	0.12***	0.16***	0.17***	0.13***	0.12***	0.10**	0.18***	0.31***	0.39***

* *<0.05*,

** *<0.01*,

*** *<0.001. PMPU, Problematic mobile phone use; BIS, Behavioral inhibition systems; BAS-R, Behavioral activation systems-reward responsiveness; BAS-D, Behavioral activation systems-drive for goal; BAS-F, Behavioral activation systems-fun seeking*.

### Network Analysis

#### Characteristics of Edges

**Figure 1:**
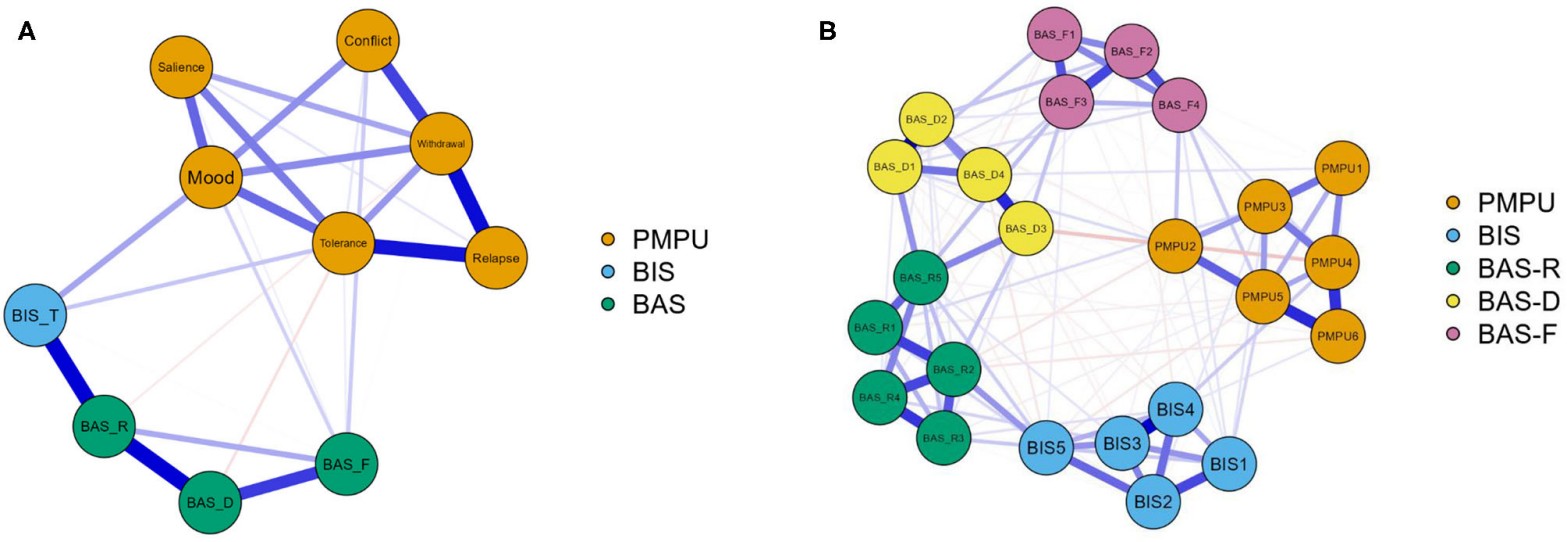
EBICglasso model based on the domain-level **(A)** and the item-level **(B)** network analysis according to the relationships between BIS, BAS-R, BAS-D, BAS-F and PMPU. PMPU, Problematic mobile phone use; BIS-T, Behavioral inhibition systems; BAS-R, Behavioral activation systems-reward responsiveness; BAS-D, Behavioral activation systems-drive for goal; BAS-F, Behavioral activation systems-fun seeking; B: PMPU1-PMPU6, Problematic mobile phone use (PMPU).

The EBICglasso network including BIS, BAS-R, BAS-D, BAS-F and six components of PMPU is presented based on the domain-level network in Figure 1A. There are 10 nodes and 27 non-zero edges in the domain-level network. Edges between nodes BIS and BAS-R and between BAS-R and BAS-D had the strongest edge intensity (*r* = 0.34 for each). In addition, nodes BIS and BAS-F had positive associations with mood modification and tolerance scores. In addition, edge weights' accuracy was relatively high and reliable based on the results of the bootstrapped network analysis (Supplementary Figure 1). The bootstrap difference test showed that most of the comparisons between edge weights were statistically significant (Supplementary Figure 2). The domain-level network is characterized by high connection strength, short paths between nodes, and overall tighter connections (SWI = 1.01).

Item-level BIS, BAS-R, BAS-D, BAS-F, and PMPU data are shown in Figure 1B. There were 22 nodes and 106 non-zero edges in the item-level network. Node BAS-F4 (“I often act on the spur of the moment”) was positively related to node PMPU2 (“Conflicts have arisen between me and my family (or friends) because of my smartphone use”; *r* = 0.09), followed by the connection between node BIS4 (“I feel pretty worried or upset when I think or know somebody is angry at me”) and node PMPU4 (“Over time, I fiddle around more and more with my smartphone”; *r* = 0.08). In addition, edge weights' accuracy was relatively accurate and reliable based on the results of the bootstrapped network analysis (Supplementary Figure 3). The bootstrap difference test showed that most of the comparisons between edge weights were statistically significant (Supplementary Figure 4). The item-level network is characterized by high connection strength, short paths between nodes, and overall tighter connections (SWI = 1.38).

#### Characteristics of Nodes

**Figure 2:**
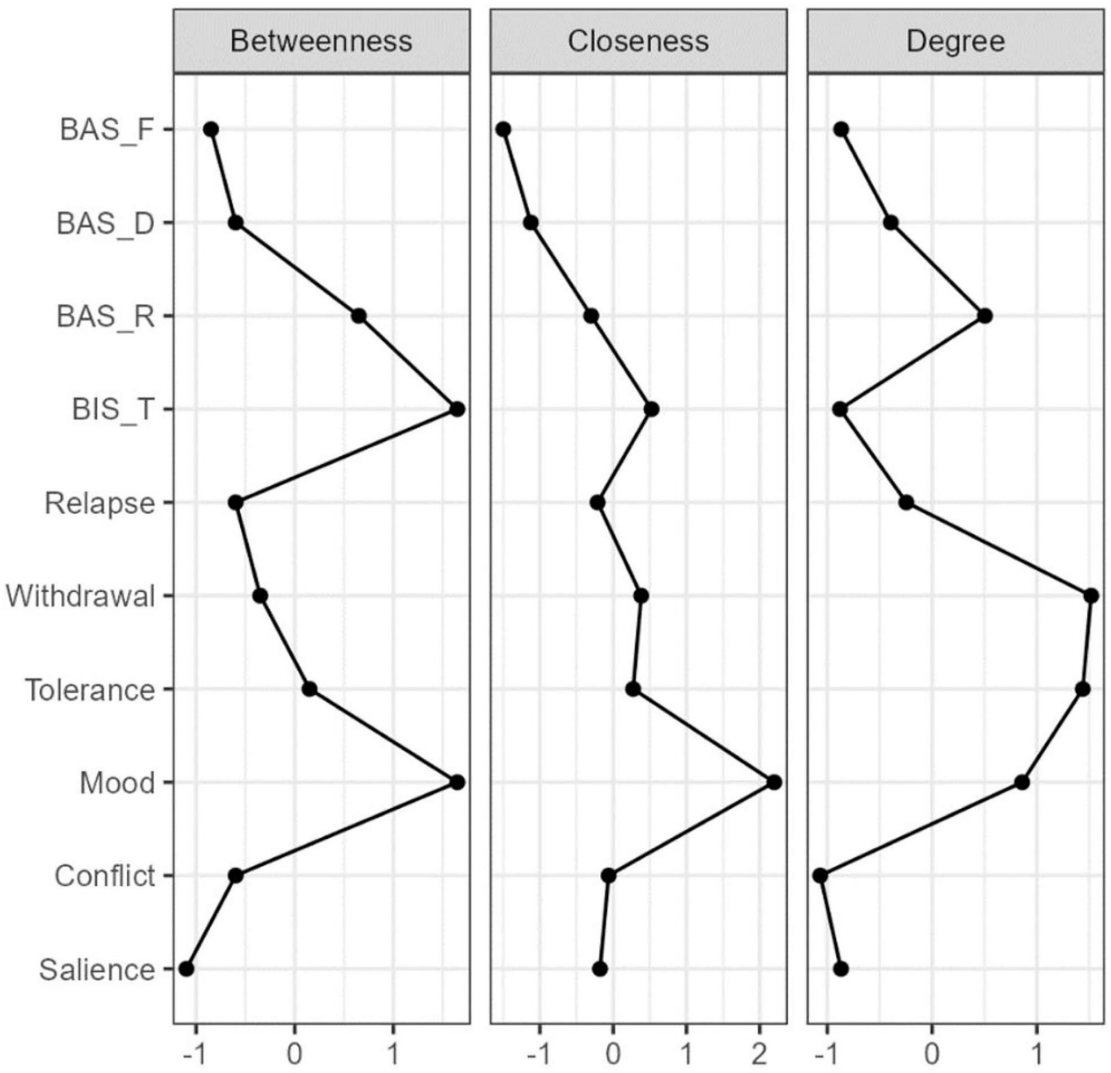
Centrality Plots for EBICglaaso network depicting the betweenness, closeness, and degree (strength), expected influence of each node (variable). BIS-T, Behavioral inhibition systems; BAS-R, Behavioral activation systems-reward responsiveness; BAS-D, Behavioral activation systems-drive for goal; BAS-F, Behavioral activation systems-fun seeking.

Table 3 and Figure 2 show the study variables' betweenness, closeness, strength (degree) and expected influence in the domain-level network. Mood modification [betweenness = 1.65 (rank = 1); closeness = 2.20 (rank = 1); strength = 0.86 (rank = 3)], BIS [betweenness = 1.65 (rank = 1); closeness = 0.52 (rank = 2); strength = −0.88 (rank = 9)], BAS-R [betweenness = 0.65 (rank = 3); closeness = −0.30 (rank = 8); strength = 0.50 (rank = 4)], tolerance (betweenness = 0.15 (rank = 4); closeness = 0.27 (rank = 4); strength = 1.44 (rank = 2)] and withdrawal symptoms [betweenness = −0.35 (rank = 4); closeness = 0.38 (rank = 3); and strength = 1.52 (rank = 1)] exhibited extremely high centrality. Among them, mood modification was the most central node. We also analyzed the stability of the network and found an excellent level of stability (i.e., *CS*-coefficient for strength = 0.67), indicating that 67% of the sample could be dropped without the network structure changing a significant extent compared to the original structure (Supplementary Figures 5, 6). The centrality stability of nodes in the network was considered to be in the preferable range (i.e., *CS*-coefficient ≥ 0.5).

**Table 3:** Centrality study variables relationship network.

	**Between**	**Closeness**	**Strength**
Salience	−1.10	−0.18	−0.87
Conflict	−0.60	−0.06	−1.07
Mood modification	1.65	2.20	0.86
Tolerance	0.15	0.27	1.44
Withdrawal symptoms	−0.35	0.38	1.52
Relapse	−0.60	−0.21	−0.25
BIS	1.65	0.52	−0.88
BAS-R	0.65	−0.30	0.50
BAS-D	−0.60	−1.12	−0.39
BAS-F	−0.85	−1.50	−0.87

*BIS, Behavioral inhibition systems; BAS-R, Behavioral activation systems-reward responsiveness; BAS-D, Behavioral activation systems-drive for goal; BAS-F, Behavioral activation systems-fun seeking*.

In the item-level network, the item PMPU3 [“Preoccupying myself with my smartphone is a way of changing my mood (I get a buzz, or I can escape or get away, if I need to)”] had higher levels of betweenness, closeness, and strength (betweenness = 1.93; closeness = 0.94; strength = 0.70) in PMPU. Item BIS4 (“I feel pretty worried or upset when I think or know somebody is angry at me”) had a higher level of strength (strength = 0.70) in BIS, and item BIS5 (“I feel worried when I think I have done poorly at something. I have very few fears compared to my friends”) had higher levels of betweenness and closeness (betweenness = 1.17, closeness = −0.10) in BIS. Item BAS-R5 (“When I see an opportunity for something I like, I get excited right away”) had higher levels of betweenness and closeness (betweenness = 2.88, closeness = 2.30) in BAS-R, and item BAS-R2 (“When I'm doing well at something, I love to keep at it”) had a higher level of strength (strength = 1.96) in BAS-R. Item BAS-D3 (“When I want something, I usually go all-out to get it”) had higher levels of betweenness and closeness (betweenness = 1.55, closeness = 2.09) in BAS-D, and item BAS-D1 (“If I see a chance to get something I want, I move on it right away”) had a higher level of strength (strength = 1.03) in BAS-D. Moreover, item BAS-F3 (“When I want something, I usually go all-out to get it”) had higher levels of betweenness, closeness, and strength (betweenness = 0.60; closeness =-0.56; strength = 0.002) in BAS-F (Supplementary Table 2). We also analyzed the stability of the network and found an excellent level of stability (i.e., *CS*-coefficient for strength = 0.75), indicating that 75% of the sample could be dropped without the network structure changing to a significant extent compared to the original structure (Supplementary Figures 7, 8). The centrality stability of nodes in the network was considered to be in the preferable range (i.e., *CS*-coefficient ≥ 0.5).

## Discussion

To the best of our knowledge, this is the first study to use network analysis to explore relationships between BIS/BAS and PMPU. First, this study clarified which specific symptoms of PMPU are related to BIS/BAS and identified which dimension of the BAS is related to PMPU. Second, this study also revealed core symptoms of PMPU among the components of the BIS/BAS-PMPU network. The network perspective adds novel characteristics about which symptoms of PMPU are most related to BIS/BAS and how specific symptoms interconnect with each other in the specific network, which may help in the better design of precise and effective interventions.

### Associations Between the BIS and PMPU

The domain-level network showed connections between mood modification, tolerance, and BIS. The item-level network further revealed a more detailed relationship, in which BIS4 (“I feel pretty worried or upset when I think or know somebody is angry at me”) was connected with PMPU4 (“Over time, I fiddle around more and more with my smartphone”). These findings are consistent with some previous studies that reported that individuals with high BIS are more likely to be addicted to mobile phones (47, 48).

The results indicated that individuals who were sensitive to punishment or termination of rewards were susceptible to become addicted to mobile phone. This may be because they are more prone to develop specific addictive symptoms such as mood modification and tolerance. A possible explanation for these results may be that those who score highly on BIS would be more likely to experience negative emotions [e.g., anxiety and depression; (65)], and attempt to escape into the virtual world provided by mobile phones to regulate moods (i.e., mood modification; 24; Csibi et al., (2)). Park and colleagues (38) also found that the association between the BIS and IA was mediated by anxiety and depression. This also dovetails with the view of RST (25) that BIS is usually activated and accompanied by avoidance behavior (e.g., to avoid the distress of daily life by mobile phone use). Additionally, tolerance is a result of a reinforcement process that increases the degree of mobile phone use to experience a mood-modifying effect that was initially obtained with a much smaller degree of use (24). Mood modification reflects the purpose of PMPU, while tolerance reflects the result of PMPU. Individuals with a high BIS are more likely to preoccupy themselves with mobile phones as a way of changing their moods and to increasingly fiddle with mobile phones.

### Associations Between the BAS and PMPU

The current study found that BAS-F has a relatively strong connection with mood modification and conflict in the domain-level network. The item-level network further revealed a more detailed relationship, in which BAS-F4 (“I often act on the spur of the moment”) and PMPU2 (“Conflicts have arisen between me and my family (or friends) because of my smartphone use”) had a positive connection. These findings are in line with the findings that fun seeking has been reported to be the only BAS subscale associated with IA (18, 38–40).

The results indicated that individuals who were sensitive to rewards or termination of punishment, especially the desire for novel rewarding experiences (i.e., BAS-F), more easily develop addictive symptoms such as mood modification and conflict. A possible explanation for the association between BAS-F and mood modification might be that BAS-F is associated with immediate gratification (66) and active hedonistic seeking (28). Mobile phones are tools providing individual entertainment, which might help individuals enjoy a positive experience (i.e., mood modification). Existing research has found that more hedonistic activities are undertaken on smartphones than on other technological devices (67).

The positive connection between BAS-F and conflict may partly be explained by the impulsive characteristics of BAS-F individuals. Gray (68) suggested that individuals with heightened impulsivity were more sensitive to reward signals. Empirical evidence has also revealed that fun seeking is associated with impulsivity (69). Impulsivity typically pertains to behaviors that are rash and spontaneous (70), which might lead to conflicts with family and friends. Park and colleagues (38) found that BAS-fun seeking predicted IA and was mediated by impulsivity. Thus, individuals with high BAS-F are also more likely to preoccupy themselves with mobile phones as a way of having fun, which may lead to conflict with family (or friends) because of mobile phone use.

### Core Symptoms of PMPU Among Specific Networks

A homeostatic network of mental disorders (e.g., PMPU) might be developed by interactions of symptoms that are no longer independent of each other. Among such homeostatic networks, central symptoms are more likely to activate other symptoms. Thus, central symptoms are thought to play a major role in causing the onset and/or maintenance of mental disorders [e.g., PMPU (71)]. Our results indicated that “preoccupying myself with my smartphone is a way of changing my mood” (i.e., mood modification) was one of the central symptoms that might play a key role in the development, persistence, remission, and relapse of PMPU, and another two indicators were “over time, I fiddle around more and more with my smartphone” (i.e., tolerance) and “if I cannot use or access my smartphone when I feel like, I feel sad, moody, or irritable” (i.e., withdrawal symptoms). These findings echo the compensatory internet use model (72), which suggests that people probably use mobile phones to reduce their painful emotions, which may result in overuse or even dependence on mobile phones. These three symptoms are more likely to activate other symptoms which finally leads to the onset and/or maintenance of PMPU.

Our results are different from Huang et al., (51) and (52), who reported that either loss of control or compulsive use was the core symptom of PMPU. There may be two explanations. One explanation for these results could be that the researchers used different measurement tools for PMPU. Huang et al., (51) adopted the smartphone addiction proneness scale (73) and took a single item as the symptom index in the network analysis. Andrade et al. (52) adopted a smartphone addiction inventory (74) and analyzed four dimensions (i.e., compulsive behavior, functional impairment, withdrawal, and tolerance) of node characteristics. Because different researchers adopted different addiction models, the core symptoms of PMPU were also different. Another possible explanation is that the core symptoms of PMPU may be different in specific relationship networks. From the perspective of analysis method, network analysis calculates the relationship strength of two nodes after considering other nodes (49). This means that the centrality of nodes will change with the increase in nodes. Therefore, the core symptoms of PMPU among the relationship network might be specific to the relationship [e.g., (54, 75)]. Certainly, the network specificity of core symptoms of PMPU needs further exploration.

### Limitations and Future Directions

The present study has several limitations that should be considered. One of the main limitations is the use of cross-sectional data and presentation of a static network, which unfortunately fails to provide dynamic information and causal relationships between variables. Therefore, it is important to explore the dynamic development of PMPU symptoms caused by BIS/BAS based on time series data. When a node in the symptom network is activated by BIS/BAS, it may further activate other symptoms “adjacent” to it in the network.

Moreover, the network analysis in this study incorporated only personality variables, ignoring the role of environment, cognition, and emotion [which are thought to play an important role in the development and maintenance of IA (22)], and may provide only a limited picture of the development and maintenance of PMPU symptoms. Future studies may consider the different levels of physiological, psychological, and environmental factors as part of the overall system and integrate these factors into a unified framework to examine the network of development and maintenance of PMPU symptoms.

Third, after the update of RST (76), different understandings of the BIS have been generated. This study was still based on the previous understanding of the BIS without considering the new role of the BIS. Future research can further explore the relationship between revised BIS and PMPU.

Last, this study did not specifically examine the content of mobile phone use. Previous studies have called for PMPU to investigate the specific contents of mobile phone use (77), which will help us to further compare the similarities and differences between different subtypes of PMPU. Future research can include types of mobile phone use in network analyses.

### Implications

The aforementioned findings contribute to PMPU research both theoretically and practically. Regarding to theoretical contributions, first, to our knowledge, our study is one of the first works to show the network of the relationships between BIS/BAS and PMPU. Emerging evidence that the BIS and BAS-F are connected with tolerance and mood modification, which are the core symptoms of PMPU, in the BIS/BAS-PMPU network, has been provided. Second, these findings might help us identify new ways in which PMPU forms and gain a better understanding of the components model of addiction. The traditional SEM/latent variable analysis approach focuses on the relationship between individual factors and PMPU [e.g., (45–48)], while ignoring the interaction between symptoms of PMPU and the dynamic network of how individual factors are related to specific symptoms of PMPU. Based on the network analysis approach, our study indicated that individuals with high BIS and BAS-F scores might develop mood modification and tolerance symptoms. These two sets of symptoms may activate one or more symptoms of PMPU that in turn activate another based on the strength of the edge linking them. The interaction among these symptoms may be the cause or result of PMPU. Last, these findings also offer a novel bridge between RST (25) and the components model of addiction (24). Based on a theoretical integration of the field in personality and addiction, the understanding of BIS/BAS is specifically related to what kind of PMPU symptoms are expanded upon, and a harmonious orientation to resolving the contradictory results of existing studies might be provided.

The current research also provides important practical contributions concerning the object and content of prevention. Regarding the object of prevention, our findings highlight the importance of centering on individuals with a high BIS or high BAS-F when formulating PMPU prevention for vulnerable individuals. Regarding the content of prevention, our findings suggest that when intervening in the addictive behavior of specific individuals with a high BIS or high BAS-F, it is necessary to intervene not only in their behaviors but also in their behavioral motivations, especially their motivation for emotion regulation. Inventions could replace PMPU by guiding these individuals to adopt appropriate emotional regulation methods (e.g., mindfulness-based cognitive-behavioral intervention (78). In turn, PMPU prevention may also consider increasing the punishment for mobile phone use and decreasing rewards associated with mobile phone use.

## Conclusion

The relationship between BIS/BAS and PMPU were examined by using network analysis. These results indicated that mood modification, tolerance, and withdrawal symptoms are central symptoms in the BIS/BAS-PMPU network. In addition, edges between mood modification, tolerance, and BIS and edge between mood modification, conflicts, and BAS-F bridge the BIS/BAS community and PMPU community. Furthermore, a new perspective on PMPU prevention is suggested by these findings. More specifically, practitioners developing interventions to overcome PMPU should consider aspects focusing on individuals who are sensitive to punishment and fun and their behavioral motivation.

## Data Availability Statement

The raw data supporting the conclusions of this article will be made available by the authors, without undue reservation.

## Ethics Statement

The studies involving human participants were reviewed and approved by Ethics Committee of Institutional Review Board of School of Psychology, Central China Normal University. Written informed consent from the participants' legal guardian/next of kin was not required to participate in this study in accordance with the national legislation and the institutional requirements.

## Author Contributions

LG executed the study and wrote the paper. HC and WL collaborated with the design. XC and WZ collaborated in the writing and editing of the final manuscript. All authors contributed to the article and approved the submitted version.

## Conflict of Interest

The authors declare that the research was conducted in the absence of any commercial or financial relationships that could be construed as a potential conflict of interest.

## Publisher's Note

All claims expressed in this article are solely those of the authors and do not necessarily represent those of their affiliated organizations, or those of the publisher, the editors and the reviewers. Any product that may be evaluated in this article, or claim that may be made by its manufacturer, is not guaranteed or endorsed by the publisher.
