# Supplementary material for: A Network Analysis of the Relationships Between Behavioral Inhibition/Activation Systems and Problematic Mobile Phone Use
Source: Front Psychiatry. 2022 Apr 1;13:832933. doi: 10.3389/fpsyt.2022.832933 (PMC9011098; doi:10.3389/fpsyt.2022.832933)
Supplement: Supplementary file 1 [file Data_Sheet_1.docx]

Appendix

1. Table S1. The abbreviation, mean scores, and standard deviations for per variable selected in the item-level network.
2. Table S2 Centrality measures per variable in the item-level network.
3. Figure S1. Accuracy of edge weights in domain-level network
4. Figure S2. Bootstrapped difference test for edge weights in domain-level network
5. Figure S3. Accuracy of edge weights in item-level network
6. Figure S4. Bootstrapped difference test for edge weights in item-level network
7. Figure S5. Stability of node centrality in domain-level network
8. Figure S6. Bootstrapped difference test for node strength in domain-level network
9. Figure S7. Stability of node centrality in item-level network
10. Figure S8. Bootstrapped difference test for node strength in item-level network

Table S1. The abbreviation, mean scores, and standard deviations for each variable selected in the item-level network.

| Variables | Abbreviation | M | SD |
| --- | --- | --- | --- |
| Components of PMPU |  |  |  |
| PMPU1: My smartphone is the most important thing in my life. | Salience | 3.94 | 1.33 |
| PMPU2: Conflicts have arisen between me and my family (or friends) because of my smartphone use. | Conflict | 3.37 | 1.48 |
| PMPU3: Preoccupying myself with my smartphone is a way of changing my mood (I get a buzz, or I can escape or get away, if I need to). | Mood modification | 4.11 | 1.26 |
| PMPU4: Over time, I fiddle around more and more with my smartphone. | Tolerance | 3.84 | 1.27 |
| PMPU5: If I cannot use or access my smartphone when I feel like, I feel sad, moody, or irritable. | Withdrawal symptoms | 3.13 | 1.29 |
| PMPU6: If I try to cut the time I use my smartphone, I manage to do so for a while, but then I end up using it as much or more than before. | Relapse | 3.30 | 1.25 |
| Components of BIS |  |  |  |
| BIS1: If I think something unpleasant is going to happen, I usually get pretty “worked up.” | BIS1 | 2.78 | .69 |
| BIS2: I worry about making mistakes. | BIS2 | 2.96 | .62 |
| BIS3: Criticism or scolding hurts me quite a bit. | BIS3 | 3.03 | .62 |
| BIS4: I feel pretty worried or upset when I think or know somebody is angry at me. | BIS4 | 2.98 | .65 |
| BIS5: I feel worried when I think I have done poorly at something. I have very few fears compared to my friends. | BIS5 | 2.97 | .57 |
| Components of BAS-R |  |  |  |
| BAS-R1: When I get something I want, I feel excited and energized. | BAS-R1 | 3.23 | .59 |
| BAS-R2: When I’m doing well at something, I love to keep at it. | BAS-R2 | 3.21 | .59 |
| BAS-R3: When good things happen to me, it affects me strongly. | BAS-R3 | 3.13 | .60 |
| BAS-R4: It would excite me to win a contest. | BAS-R4 | 3.22 | .56 |
| BAS-R5: When I see an opportunity for something I like, I get excited right away. | BAS-R5 | 3.15 | .62 |
| Components of BAS-D |  |  |  |
| BAS-D1: When I want something, I usually go all-out to get it. | BAS-D1 | 2.78 | .67 |
| BAS-D2: I go out of my way to get things I want. | BAS-D2 | 2.90 | .64 |
| BAS-D3: If I see a chance to get something I want, I move on it right away. | BAS-D3 | 2.92 | .60 |
| BAS-D4: When I go after something I use a “no holds barred” approach. | BAS-D4 | 2.66 | .68 |
| Components of BAS-F |  |  |  |
| BAS-F1: I will often do things for no other reason than that they might be fun. | BAS-F1 | 2.82 | .64 |
| BAS-F2: I crave excitement and new sensations. | BAS-F2 | 2.83 | .66 |
| BAS-F3: I'm always willing to try something new if I think it will be fun. | BAS-F3 | 2.93 | .60 |
| BAS-F4: I often act on the spur of the moment. | BAS-F4 | 2.59 | .73 |

Note. PMPU= Problematic mobile phone use, BIS =Behavioral inhibition systems, BAS-R= Behavioral activation systems-reward responsiveness, BAS-D= Behavioral activation systems-drive for goal, BAS-F= Behavioral activation systems-fun seeking.

Table S2 Centrality measures per variable in the item-level network.

|  | | **Network** | | | | | |
| --- | --- | --- | --- | --- | --- | --- | --- |
| **Variable** | | **Betweenness** | | **Closeness** | | **Strength** | |
| PMPU1 |  | -1.046 |  | -1.309 |  | -2.292 |  |
| PMPU2 |  | -0.667 |  | -0.602 |  | -1.412 |  |
| PMPU3 |  | 1.926 |  | 0.942 |  | 0.698 |  |
| PMPU4 |  | -0.287 |  | -0.012 |  | -1.193 |  |
| PMPU5 |  | -0.667 |  | -0.369 |  | -0.418 |  |
| PMPU6 |  | 0.029 |  | -0.137 |  | 1.509 |  |
| BIS1 |  | -0.603 |  | -0.952 |  | -0.799 |  |
| BIS2 |  | -0.603 |  | -0.510 |  | 0.162 |  |
| BIS3 |  | -0.983 |  | -0.980 |  | 0.480 |  |
| BIS4 |  | -0.098 |  | -1.103 |  | 0.707 |  |
| BIS5 |  | 1.167 |  | -0.099 |  | -0.072 |  |
| BAS-R1 |  | -0.287 |  | 1.478 |  | -0.870 |  |
| BAS-R2 |  | 0.535 |  | 0.924 |  | 1.963 |  |
| BAS-R3 |  | -0.730 |  | -0.320 |  | 0.236 |  |
| BAS-R4 |  | -0.098 |  | 0.807 |  | -0.278 |  |
| BAS-R5 |  | 2.875 |  | 2.296 |  | 1.487 |  |
| BAS-D1 |  | -0.034 |  | 0.542 |  | 1.025 |  |
| BAS-D2 |  | -0.856 |  | 0.047 |  | 0.137 |  |
| BAS-D3 |  | 1.547 |  | 2.087 |  | 0.658 |  |
| BAS-D4 |  | -0.793 |  | 0.425 |  | -0.083 |  |
| BAS-F1 |  | -0.920 |  | -1.081 |  | -1.148 |  |
| BAS-F2 |  | -0.098 |  | -0.703 |  | -0.447 |  |
| BAS-F3 |  | 0.598 |  | -0.555 |  | 0.002 |  |
| BAS-F4 |  | 0.092 |  | -0.815 |  | -0.052 |  |

Note. PMPU= Problematic mobile phone use, BIS =Behavioral inhibition systems, BAS-R= Behavioral activation systems-reward responsiveness, BAS-D= Behavioral activation systems-drive for goal, BAS-F= Behavioral activation systems-fun seeking, Cluster=signed Zhang clustering coefficient.


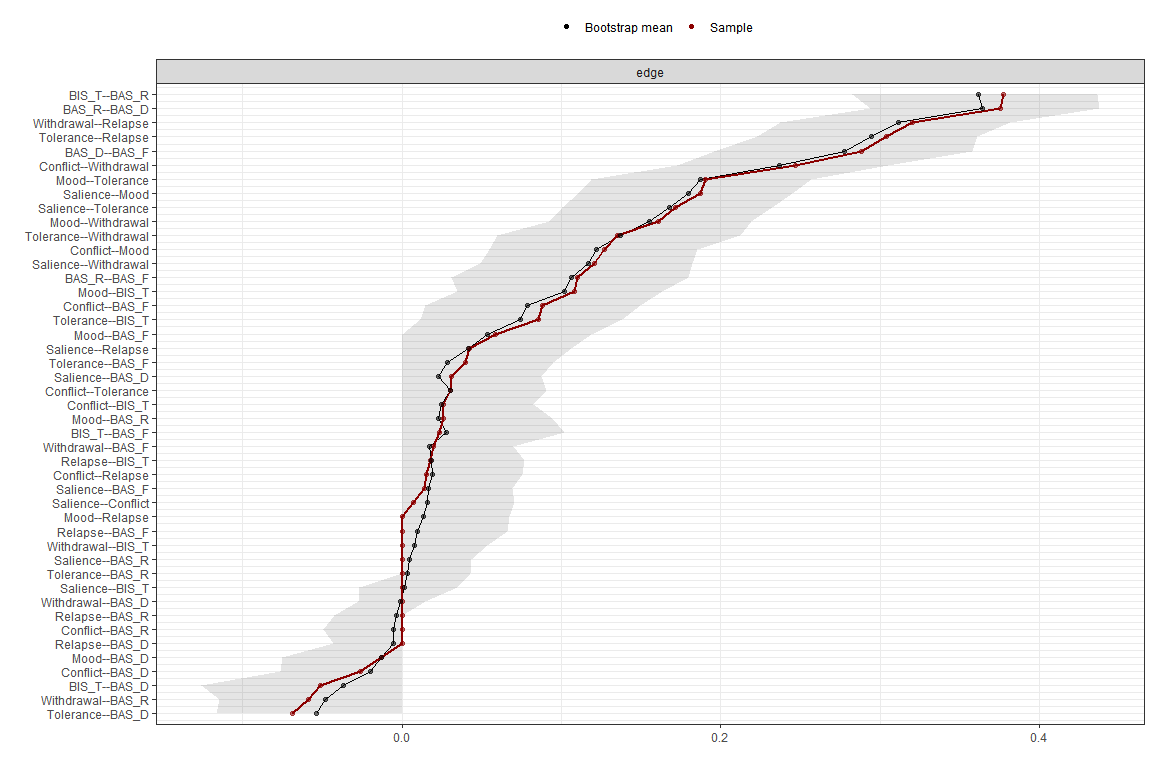


Figure S1. Accuracy of edge weights in domain-level network

Note: The red line depicts the sample edge weights and the gray bar depicts the bootstrapped confidence interval.


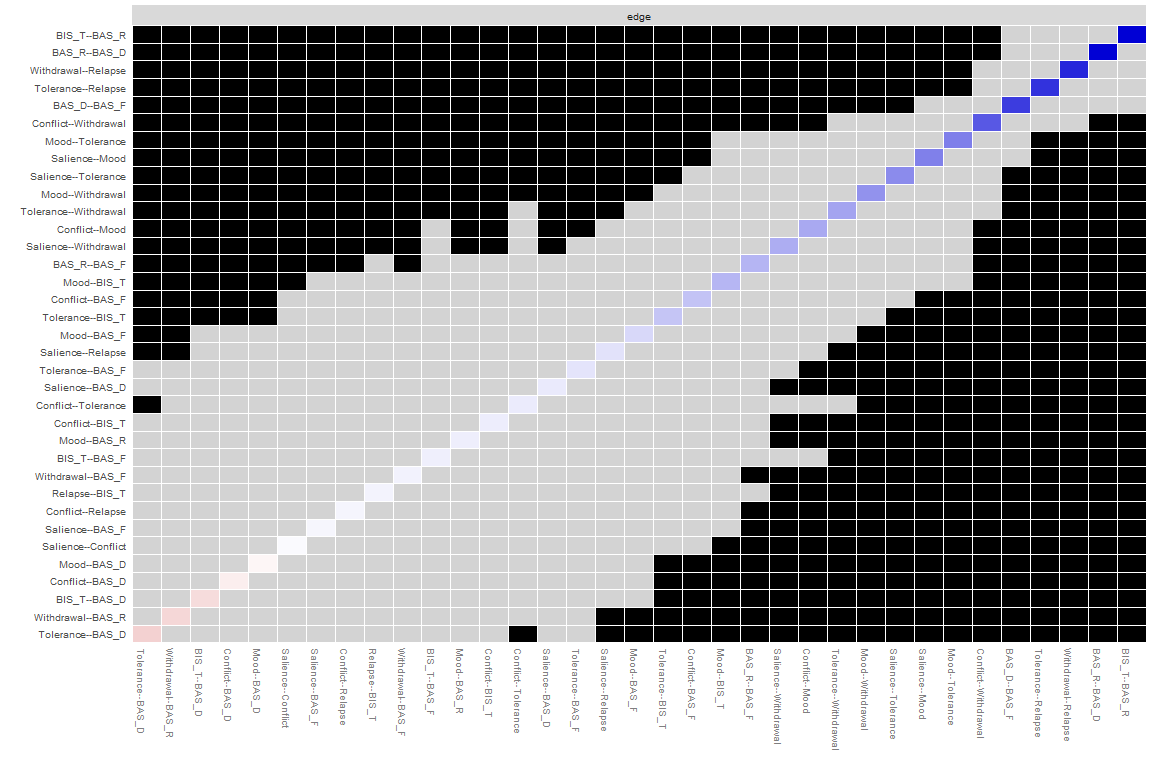


Figure S2. Bootstrapped difference test for edge weights in domain-level network

Note: Gray boxes indicate edge weights that do not differ significantly from one another, while black boxes indicate edge weights that do differ significantly. Blue and red boxes on the diagonal correspond to edge weights with positive and negative correlations, respectively.


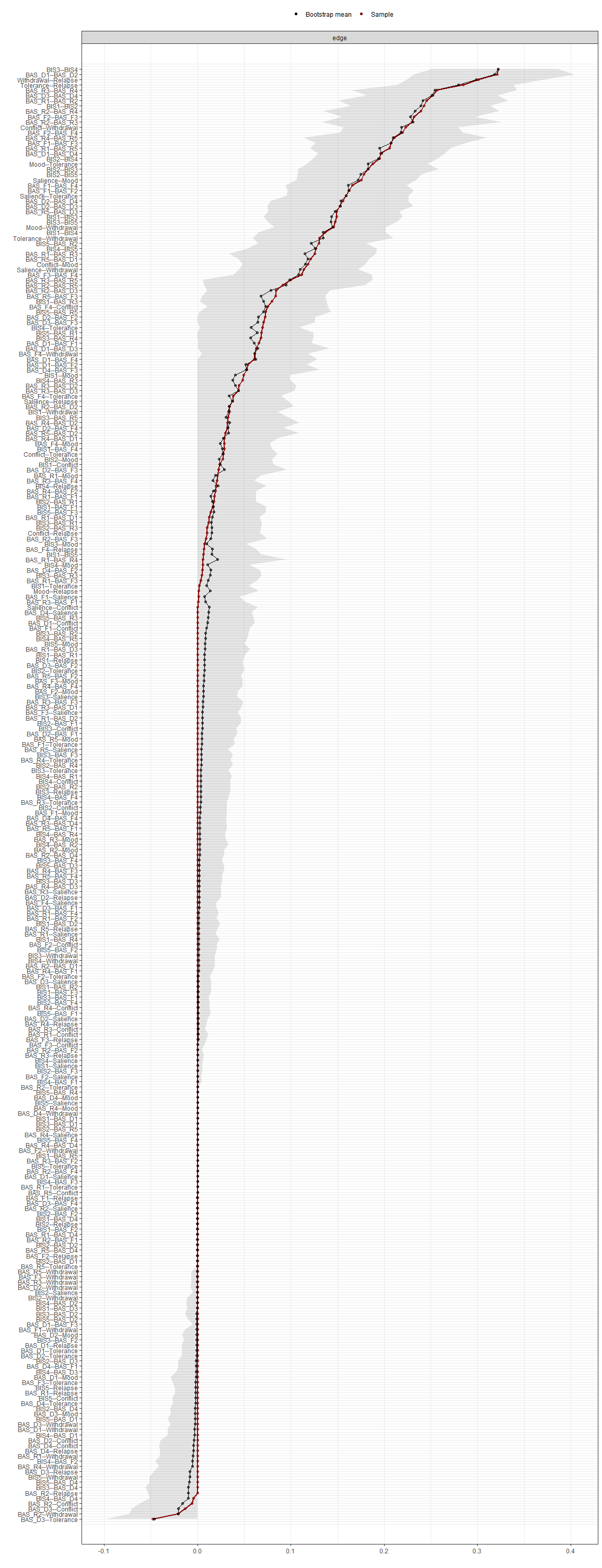


Figure S3. Accuracy of edge weights in item-level network

Note: The red line depicts the sample edge weights and the gray bar depicts the bootstrapped confidence interval.


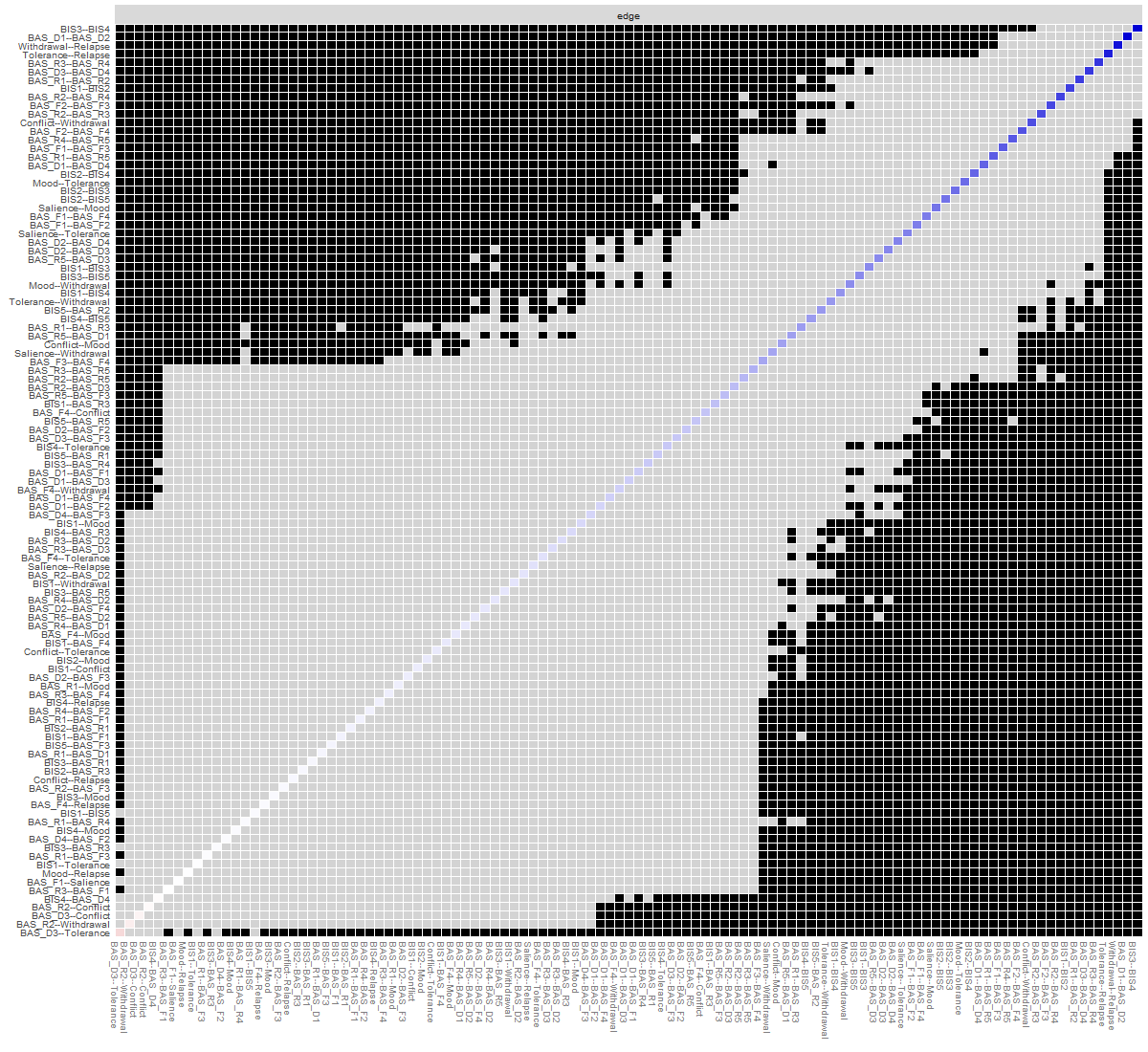
Figure S4. Bootstrapped difference test for edge weights in item-level network

Note: Gray boxes indicate edge weights that do not differ significantly from one another, while black boxes indicate edge weights that do differ significantly. Blue and red boxes on the diagonal correspond to edge weights with positive and negative correlations, respectively.


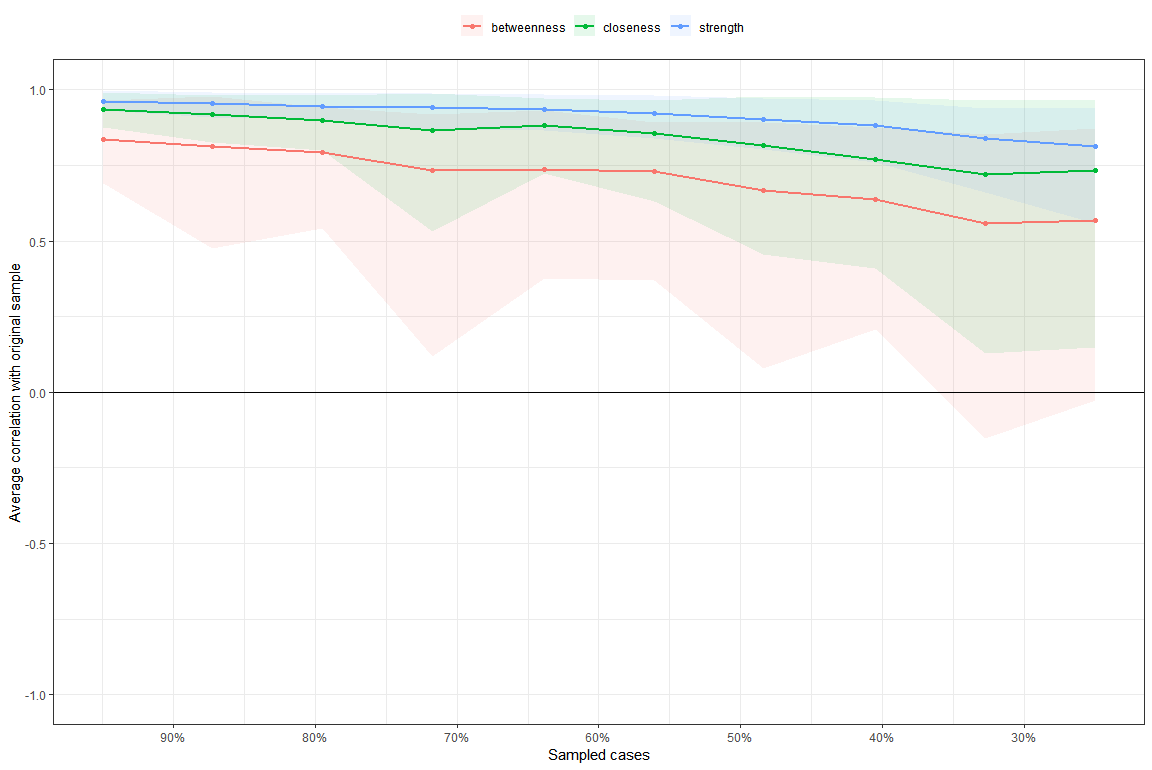


Figure S5. Stability of node centrality in domain-level network

Note: Average correlation between node strength, closeness and strength sampled with persons dropped and the original sample. Red, green or blue lines and areas respectively indicated the means and the range from the 2.5th quantile to the 97.5th quantile.


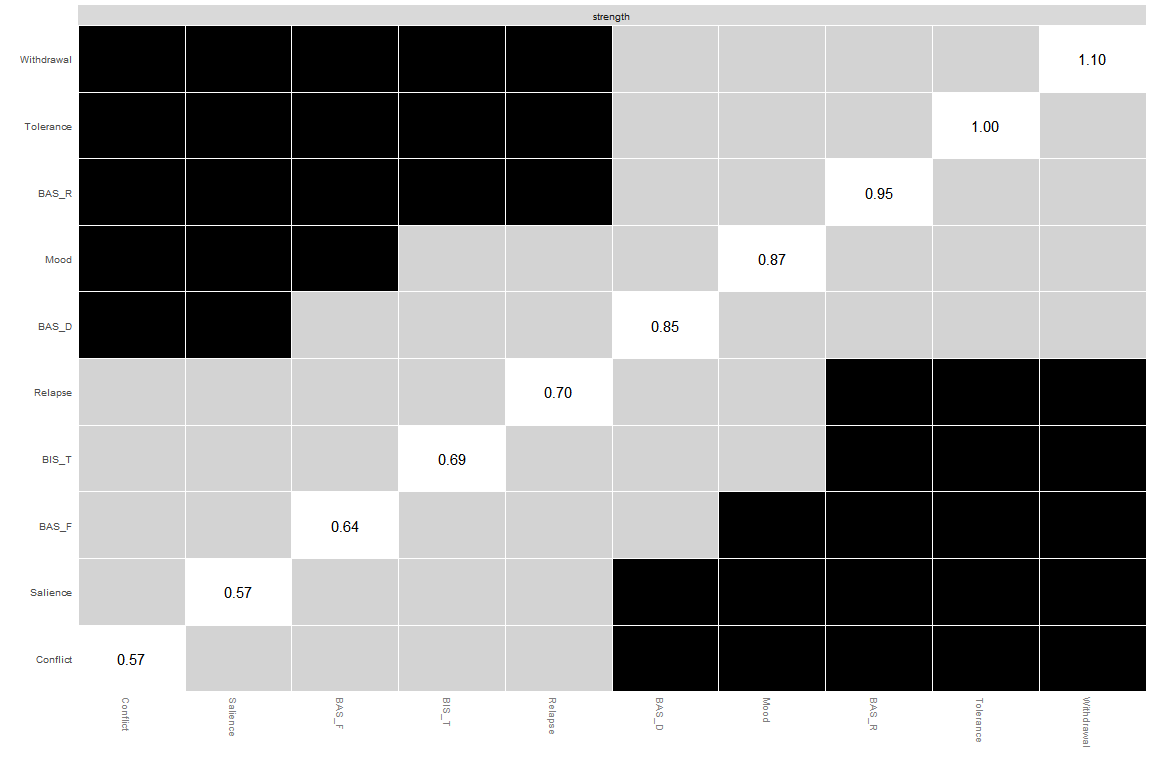


Figure S6. Bootstrapped difference test for node strength in domain-level network

Note: Gray boxes indicate node strength that do not differ significantly from one another, while black boxes indicate node strength that do differ significantly. The number in the white boxes (i.e., diagonal line) represent the value of node strength.


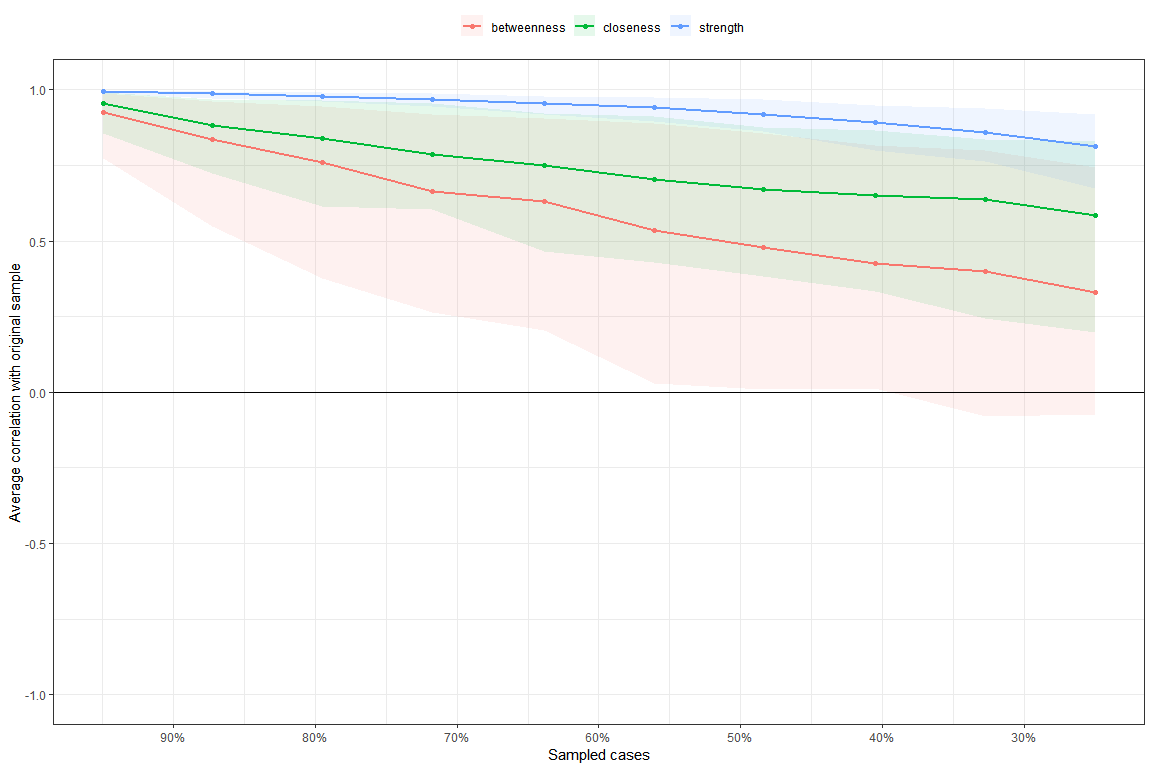


Figure S7. Stability of node centrality in item-level network

Note: Average correlation between node strength, closeness and strength sampled with persons dropped and the original sample. Red, green or blue lines and areas respectively indicated the means and the range from the 2.5th quantile to the 97.5th quantile.


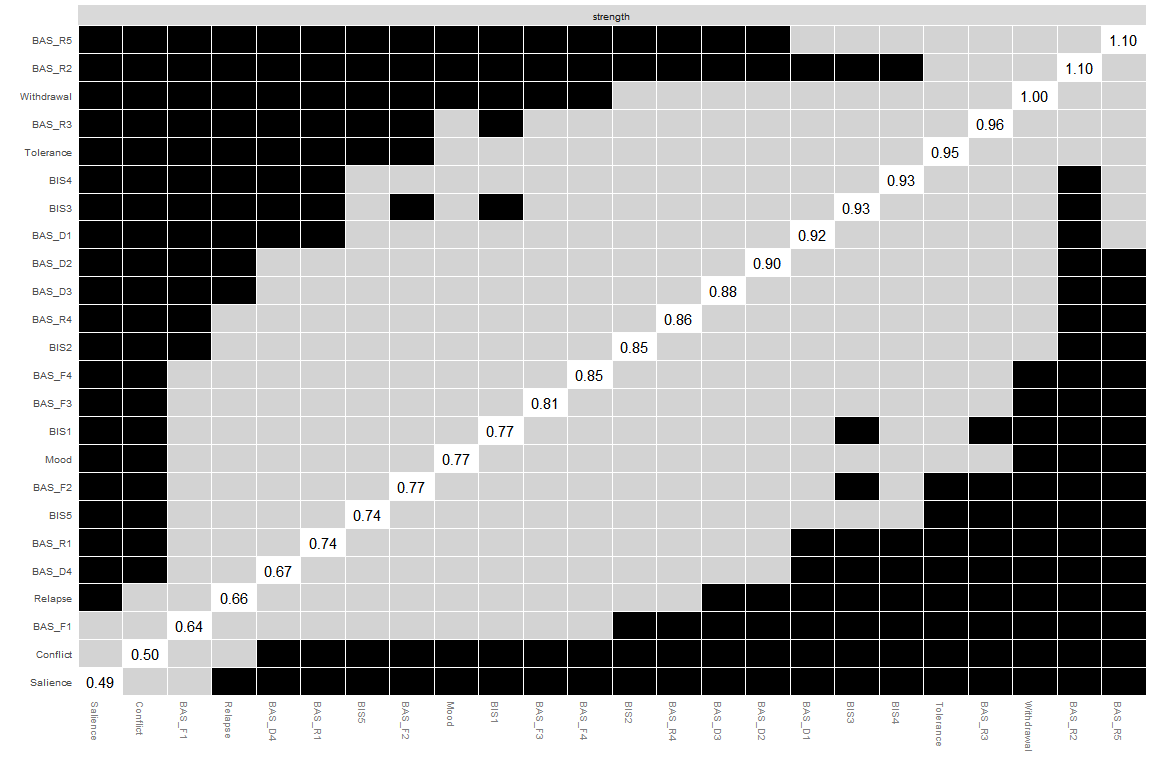


Figure S8. Bootstrapped difference test for node strength in item-level network

Note: Gray boxes indicate node strength that do not differ significantly from one another, while black boxes indicate node strength that do differ significantly. The number in the white boxes (i.e., diagonal line) represent the value of node strength.
